# Is Prior Zika Virus Infection Associated With Cardiovascular Disease?

**DOI:** 10.7759/cureus.47141

**Published:** 2023-10-16

**Authors:** Diego Vasquez, Ana Palacio, Peter Chedraui, Maria Del Mar Sanchez, Wladimir Briones, Leonardo Tamariz, Marco A Calle

**Affiliations:** 1 Department of Epidemiology, Universidad Católica de Santiago de Guayaquil, Guayaquil, ECU; 2 Population Health and Computational Medicine, University of Miami Miller School of Medicine, Miami, USA; 3 Escuela de Posgrado en Salud, Universidad Espíritu Santo, Samborondón, ECU; 4 Department of Public Health and Epidemiology, Universidad Católica de Santiago de Guayaquil, Guayaquil, ECU; 5 Department of Epidemiology, Ministerio de Salud Publica, Quito, ECU; 6 Department of Medicine, Universidad Católica de Santiago de Guayaquil, Guayaquil, ECU

**Keywords:** valvular regurgitation, cardiac arrhythmias (ca), left ventricular hypertrophy (lvh), diastolic dysfunction, 24-hour holter, echocardiogram (echo), cardio vascular disease, zika diagnosis, zika infection

## Abstract

Background: Zika virus (ZIKV) infection is associated with severe complications. Recently, reports have raised the possibility of cardiovascular complications. However, the complications that are reported are in case reports and occur immediately after infection. Our aim is to evaluate the cardiovascular complications of ZIKV infection in a younger patient population.

Methods: We conducted a prospective cohort and included patients with a one-year history of prior confirmed ZIKV infection. We performed an echocardiogram, a 24-hour automated blood pressure, and a 24-hour Holter. Our primary outcome included a composite of having diastolic dysfunction, left ventricular hypertrophy, arrhythmias, valvular regurgitation, premature beats, or non-dipper status.

Results: We included 47 patients with ZIKV and 16 patients without ZIKV. Patients with ZIKV had a similar age as controls (p>0.05). Having had a prior ZIKV infection was associated with diastolic dysfunction, left ventricular hypertrophy, valvular regurgitation, arrhythmias or premature beats, and non-dipper status (p<0.05). The adjusted OR of having the primary outcome was 2.3; 95% CI 1.3-2.7. After one year, IL-10 and C-reactive protein (CRP) were higher in ZIKV-infected patients compared to controls (p<0.05).

Conclusions: Our study found that young patients with a prior ZIKV infection have more echocardiographic, arrhythmic, and blood pressure changes when compared to similar-aged controls.

## Introduction

Zika virus (ZIKV) is a positive-sense single-stranded RNA arbovirus that belongs to the genus Flavivirus of the family Flaviviridae. The current surge of ZIKV has been perpetuated by climate change [1], natural disasters [2], and new modes of transmission (mother-to-fetus, blood products, and sexual transmission) [1].

An unexpected and striking feature of the ZIKV pandemic was the emergence of complications. Clusters of Guillain-Barre syndrome and an increase in incident cases of microcephaly were attributed to ZIKV infection [3]. A recently emerged potential complication is cases of different cardiovascular diseases (CVD).

Other infections have been linked to CVD. Endemic areas of dengue and Chikungunya have reported cases of CVD, particularly heart failure. The mechanism of arboviral CVD complications could be a direct viral invasion of the heart muscle [4]. Several case reports have identified acutely infected ZIKV patients with myocarditis and atrial fibrillation [5,6]. However, another potential mechanism for ZIKV-related CVD could be an acute viral infection leading to chronic inflammation and developing CVD over time [7]. Therefore, our aim was to evaluate the development of incident CVD several years after a ZIKV infection and determine if having cardiovascular symptoms is associated with CVD.

## Materials and methods

Study design and population

We conducted a prospective cohort study and included two groups of patients. One group was a sample of patients who were diagnosed with ZIKV using PCR by the Ministry of Health in 2017 within three municipalities in Ecuador, and another was a group of concurrent controls without ZIKV infection. The study was approved by the Subsistema de Investigación y Desarrollo de la Universidad Católica de Santiago de Guayaquil (SINDE) research ethics board (SINDE-01222-2017), and all subjects who participated in the study signed informed consent.

Definition of ZIKV infection

We defined incident ZIKV cases as those with a positive laboratory result for ZIKV. The testing in Ecuador was performed by the Ministry of Health using CFX96 and CFX384 real-time RT-PCR systems (Bio-Rad Laboratories, Invitrogen, Hercules, CA). We used the oligonucleotides ZIKV 1087/ZIKV 1163 and the ZIKV-FAM probe in a final volume of 25 µl, according to the Lanciotti protocol [8]. All positive ZIKV cases were reported into a registry that we have used in the past for research [9]. We only included ZIKV-confirmed cases in one geographical area because participants had to attend a cardiology clinic we partnered with for CVD testing. Within the ZIKV group, we selected a random sample of 50 patients, 25 with CVD symptoms and 25 without them. We used a validated survey to evaluate cardiovascular symptoms [10]. The rationale for including a group with symptoms is to determine if those patients have a higher probability of having CVD. We excluded patients with a prior self-report of hypertension, diabetes, coronary artery disease, atrial fibrillation, or heart failure [11]. The validity of cardiovascular risk factor self-reports has been previously documented [12].

Definition of controls

Controls were obtained from the family members of the ZIKV-positive patients. To be included as controls, patients needed to have no symptoms of ZIKV infection, no prior self-report of CVD, and be of similar age.

Cardiovascular evaluation

We performed three cardiovascular tests on all participants: an echocardiogram, a 24-hour Holter, and 24-hour blood pressure monitoring. Testing was performed approximately two years after the ZIKV infection. We also measured interleukin-10 (IL-10) and C-reactive protein (CRP), as both have been associated with cardiovascular diseases [13]. The echocardiogram was performed according to American Society of Echocardiography guidelines [14]. The echocardiogram evaluated diastolic dysfunction, left ventricular mass, and ejection fraction. The ambulatory blood pressure (ABPM) was measured with a validated and calibrated Sony device. The monitor was placed on the participants’ nondominant arm, measured BP every 30 minutes, and was set so that readings were not displayed. The AMBP evaluated daytime and nighttime blood pressure and determined dipper and non-dipper status. The 24-hour Holter monitoring was performed simultaneously with the 24-hour ABPM. Time was synchronized between the two devices before each application. The 24-hour Holter evaluated arrhythmias. A cardiologist blinded to ZIKV status interpreted all three tests.

Primary outcome

Our primary outcome included a composite of having diastolic dysfunction, left ventricular hypertrophy, arrhythmias, valvular regurgitation, or non-dipper status. We defined diastolic dysfunction as grade I (impaired relaxation pattern), grade II (pseudonormalized filling), and grade III (restrictive pattern) [15]. We defined left ventricular hypertrophy as any abnormal left ventricular size measurement (posterior or septal thickness >0.9 cm in women or >1.0 cm in males) or a left ventricular mass index >95 g/m^2^ in women or >115 g/m^2^ in males [16].

We defined arrhythmias as premature beats or persistent sinus bradycardia or tachycardia. We defined valvular regurgitation as mild regurgitation on any valve and non-dipper status as a drop in nighttime blood pressure of <10% [17].

Statistical analysis

We evaluated the distribution of the continuous variables using measures of central tendency and skewness. We compared baseline characteristics by ZIKV status using chi-square and t-tests.

Comparisons of the outcome between both groups were also done with chi-square. We conducted a multivariate analysis to evaluate if having ZIKV was associated with having the primary outcome. We used logistic regression to calculate the odds ratio (OR) and corresponding 95% confidence interval (CI) adjusted for age and gender. To evaluate if symptoms were associated with a CVD finding, we used dummy variables for those with ZIKV infection with and without symptoms and used controls as the reference.

The fitness of the data was assessed using the deviance ratio. Analyses were performed using the STATA version (College Station, Texas), and all significance tests were two-tailed.

## Results

Baseline characteristics

In 2017, there were 390 subjects with a positive ZIKV assay in the three municipalities where the study was conducted. Table 1 presents the baseline characteristics of the 63 included patients. We included 47 patients with ZIKV with a mean number of days after diagnosis of 1.25±0.07 years and 16 patients without ZIKV. The mean age of patients with ZIKV was 31.9±18.2 and was similar to that of controls (p>0.05). Also, ZIKV patients were more likely to be female (85%) when compared to controls (44%; p=0.01).

**Table 1:** Baseline characteristics ZIKV: Zika virus

Characteristic	ZIKV positive patients	ZIKV negative patients	p-value
Number	47	16	
Mean age	31.9±18.2	29.1±12.2	0.57
Female sex, %	85%	44%	0.01

Association between ZIKV infection and CV testing

Table 2 presents the association between ZIKV and CVD. The primary outcome was more common among ZIKV patients (100%) compared to 25% of those without ZIKV (25%) (p<0.01). When analyzed individually, diastolic dysfunction, left ventricular hypertrophy, valvular regurgitation, and non-dipper status were significantly more frequent among ZIKV-infected patients when compared to controls (p<0.01). One patient had grade II diastolic dysfunction, one had atrial fibrillation, and one had an atrioventricular block; all had a prior ZIKV infection. All valvular regurgitation was mild and had no hemodynamic significance. The adjusted OR of having the primary outcome was 2.3; 95% CI 1.3-2.7.

**Table 2:** Association between Zika virus infection and cardiovascular diseases CVD: cardiovascular disease, ZKV: Zika virus

CVD outcome	ZIKV positive patients	ZIKV negative patients	p-value
Diastolic dysfunction, %	20%	0%	<0.01
Left ventricular hypertrophy, %	9%	0%	<0.01
Valvular regurgitation, %	100%	50%	<0.01
Premature ventricular contraction, %	33%	13%	0.17
Non-dipper status, %	26%	0%	<0.01

Association between CVD symptoms and ZIKV infection

Table 3 shows the selected CVD work-up and ZIKV status. We recruited 24 patients with ZIKV infection and CVD symptoms and 23 with ZIKV and no CVD symptoms. Having symptoms was associated with a higher probability of the primary outcome (100%) for those with CVD symptoms and ZIKV than those without symptoms (100%).

**Table 3:** Symptoms and Zika virus infection CVD: cardiovascular diseases, ZIKV: Zika virus

CVD outcome	ZIKV positive with symptoms	ZIKV positive without symptoms	Controls	p-value
Number	24	23	16	
Mean age	34.0±20.3	29.7±12.2	29.1±12.2	0.11
Female, %	96%	74%	75%	<0.01
Primary outcome, %	100%	100%	25%	<0.01
Diastolic dysfunction, %	21%	18%	0%	0.42
Left ventricular hypertrophy, %	13%	4%	0%	0.41

Biomarkers and ZIKV infection

**Figure 1:**
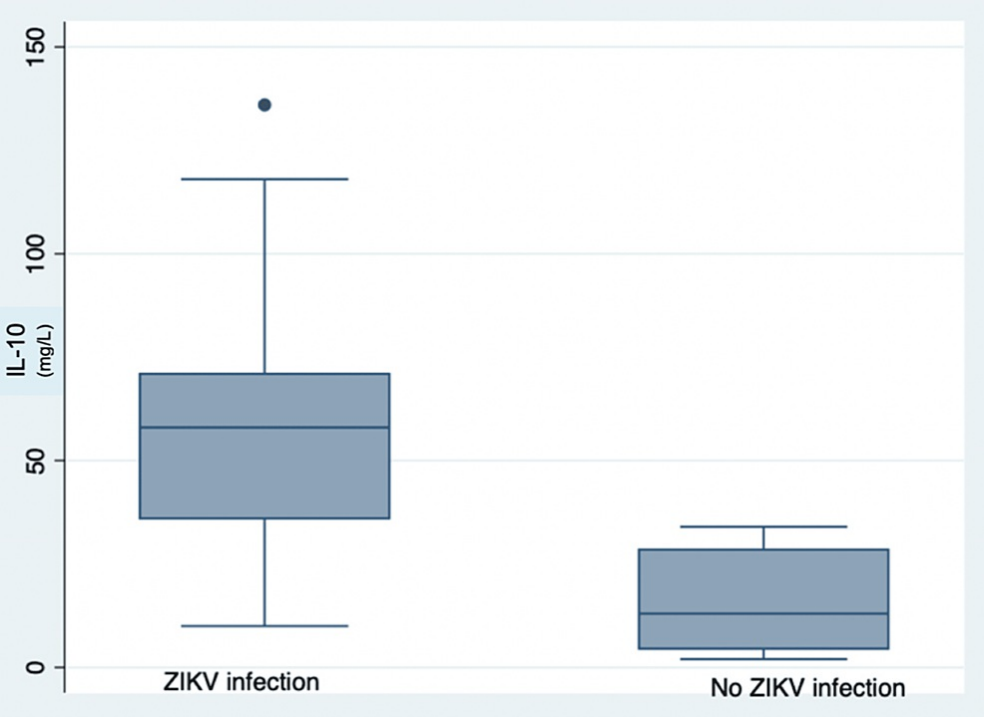
Relation between Zika virus infection and interleukin-10 levels IL-10: interleukin-10, ZIKV: Zika virus

**Figure 2:**
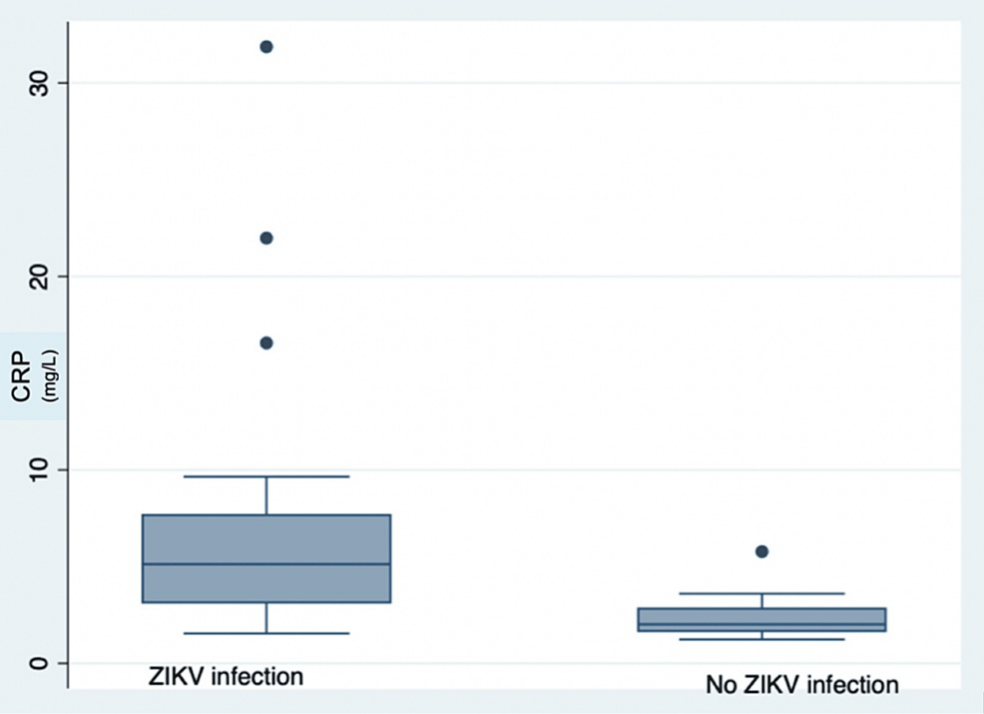
Relation between Zika virus infection and C-reactive protein levels ZIKV: Zika virus, CRP: C-reactive protein

Figures 1-2 show the differences in both IL-10 and CRP by Zika status. Having ZIKV was associated with higher levels of IL-10 and CRP compared to controls (P<0.05). There was no difference in the level of IL-10 and CRP between those with and without CVD symptoms (p>0.05).

## Discussion

Our study found that young patients with a one-year history of prior ZIKV infection and no prior CVD have more echocardiographic, arrhythmic, and blood pressure changes when compared to similar-aged controls. At the same time, those with ZIKV had a higher level of inflammatory markers than controls. The strengths of this study when compared to other studies are the use of a control population, the use of a population with a prior history of ZIKV rather than acute infection, and the measurement of inflammatory markers as a potential mechanism of CVD [18].

Several studies have already pointed towards an association between ZIKV and heart disease. These have reported an association between ZIKV acute infection and atrial fibrillation [19] and myocarditis [6,20] among infected adults living in endemic areas. A case series of nine patients without prior heart disease who presented with heart-related complaints underwent holter, cardiac MRI, and echocardiogram. The study found that eight patients had arrhythmias and six presented with heart failure [5]. However, these studies are limited by their small sample size, lack of a control group, and the evaluation of CVD immediately after ZIKV infection.

Our study found a higher prevalence of subclinical CVD one year post-ZIKV infection in a young population without heart disease or prior risk factors when compared to a control group. We evaluated inflammation as a possible mechanism for the development of CVD related to ZIKV. The role of inflammation in prognosis is the subject of recent studies, as an early immune response to ZIKV infection may determine its clinical manifestations and outcome, including neurological and cardiovascular. Others have already compared the inflammatory response in those with ZIKV and controls [21], and different interleukins were associated with specific ZIKV symptoms. In our study, we found that those with ZIKV and CVD had higher inflammatory markers than those without ZIKV. This is of particular importance since interleukins have been associated with heart failure and coronary artery disease [13], suggesting these patients may be at a higher CVD risk even years post-infection. Our study also allows us to evaluate if the presence of symptoms could identify ZIKV patients at CVD risk, and our findings suggest it does not since all asymptomatic ZIKV patients had at least one of the CVD outcomes included in the primary outcome.

Our study has several limitations that deserve mention. First, our sample size, even though it is larger than prior studies, is still small, particularly for the control group. However, with a small sample size, we were able to detect statistical significance. Second, we cannot prove causality since we did not perform cardiac magnetic resonance imaging or myocardial biopsies to detect viral replication. Third, our groups were not completely matched, and we could only report limited multivariate models adjusting for all risk factors related to heart disease because of the small sample size.

A larger prospective cohort is warranted to better evaluate the effects of a prior ZIKV infection on CVD outcomes and establish evidence-based guidelines for CVD monitoring post-ZIKV infection.

## Conclusions

In conclusion, our study provides further scientific evidence that ZIKV could be associated with future subclinical CVD in the year following the infection, even among patients without symptoms, as evidenced by more alterations in 24-hour Holter, 24-hour blood pressure monitoring, and echocardiography in young patients with ZIKV infection compared to the control group. Due to the scarcity of studies in Latin America, we consider it important to carry out more studies related to this topic for a greater understanding of the relationship and causality of ZIKV and CVD.

## References

[1] Rossi SL, Estofolete CF, Nogueira ML, Vasilakis N (2018). Age and sex in the zika pandemic era. J Infect Dis.

[2] Baud D, Gubler DJ, Schaub B, Lanteri MC, Musso D (2017). An update on Zika virus infection. Lancet.

[3] Musso D, Roche C, Robin E, Nhan T, Teissier A, Cao-Lormeau VM (2015). Potential sexual transmission of Zika virus. Emerg Infect Dis.

[4] Guanche Garcell H, Gutiérrez García F, Ramirez Nodal M, Ruiz Lozano A, Pérez Díaz CR, González Valdés A, Gonzalez Alvarez L (2020). Clinical relevance of Zika symptoms in the context of a Zika Dengue epidemic. J Infect Public Health.

[5] Minhas AM, Nayab A, Iyer S, Narmeen M, Fatima K, Khan MS, Constantin J (2017). Association of Zika virus with myocarditis, heart failure, and arrhythmias: a literature review. Cureus.

[6] Zambrano LI, Rodriguez E, Espinoza-Salvado IA, Rodríguez-Morales AJ (2019). Dengue in Honduras and the Americas: the epidemics are back!. Travel Med Infect Dis.

[7] Pothineni NV, Subramany S, Kuriakose K, Shirazi LF, Romeo F, Shah PK, Mehta JL (2017). Infections, atherosclerosis, and coronary heart disease. Eur Heart J.

[8] Vernocchi P, Maffei M, Lanciotti R, Suzzi G, Gardini F (2007). Characterization of Mediterranean mussels (Mytilus galloprovincialis) harvested in Adriatic Sea (Italy). Food Control.

[9] Vasquez D, Palacio A, Nuñez J, Briones W, Beier JC, Pareja DC, Tamariz L (2017). Impact of the 2016 Ecuador earthquake on Zika virus cases. Am J Public Health.

[10] Ramacciotti E, Agati LB, Aguiar VC (2019). Zika and chikungunya virus and Risk for venous thromboembolism. Clin Appl Thromb Hemost.

[11] Bandyopadhyay D, Ashish K, Ghosh S, Hajra A, Modi VA (2018). Cardiovascular implications of Zika virus infection. Eur J Intern Med.

[12] Dey AK, Alyass A, Muir RT, Black SE, Swartz RH, Murray BJ, Boulos MI (2015). Validity of self-report of cardiovascular risk factors in a population at high risk for stroke. J Stroke Cerebrovasc Dis.

[13] Shim BS, Kwon YC, Ricciardi MJ (2019). Zika virus-immune plasmas from symptomatic and asymptomatic individuals enhance Zika pathogenesis in adult and pregnant mice. mBio.

[14] Cavalcanti DD, Alves LV, Furtado GJ (2017). Echocardiographic findings in infants with presumed congenital Zika syndrome: Retrospective case series study. PLoS One.

[15] Liu H, Zhou W, Liao H, Hu Z, Zou M, Liu S (2019). [A non-coated enzyme-linked immunosorbent assay for screening zika virus envelope protein]. Nan Fang Yi Ke Da Xue Xue Bao.

[16] de Simone G, Mancusi C, Izzo R, Losi MA, Aldo Ferrara L (2016). Obesity and hypertensive heart disease: focus on body composition and sex differences. Diabetol Metab Syndr.

[17] Lonati L, Cuspidi C, Sampieri L, Boselli L, Bocciolone M, Leonetti G, Zanchetti A (1992). Prevalence of physiological valvular regurgitation in hypertensive patients: echocardiographic and color Doppler study. Cardiology.

[18] Nutho B, Rungrotmongkol T (2019). Binding recognition of substrates in NS2B/NS3 serine protease of Zika virus revealed by molecular dynamics simulations. J Mol Graph Model.

[19] Abdalla LF, Santos JH, Barreto RT (2018). Atrial fibrillation in a patient with Zika virus infection. Virol J.

[20] Aletti M, Lecoules S, Kanczuga V, Soler C, Maquart M, Simon F, Leparc-Goffart I (2017). Transient myocarditis associated with acute Zika virus infection. Clin Infect Dis.

[21] Adachi K, Romero T, Nielsen-Saines K (2020). Early clinical infancy outcomes for microcephaly and/or small for gestational age Zika-exposed infants. Clin Infect Dis.

